# Oxidative Balance Score Is Associated with Social Involvement and Weight-Adjusted Appendicular Skeletal Muscle Mass in Middle-Aged and Older Japanese Women

**DOI:** 10.3390/nu18040688

**Published:** 2026-02-20

**Authors:** Tamami Odai, Masakazu Terauchi, Yuka Enokuchi, Naoyuki Miyasaka

**Affiliations:** 1 Department of Women’s Health, Institute of Science Tokyo, Yushima 1-5-45, Bunkyo, Tokyo 113-8510, Japan; odycrm@tmd.ac.jp (T.O.);; 2 Department of Obstetrics and Gynecology, Institute of Science Tokyo, Yushima 1-5-45, Bunkyo, Tokyo 113-8510, Japan; n.miyasaka.gyne@tmd.ac.jp

**Keywords:** muscle health, oxidative stress, social engagement, well-being

## Abstract

Background: The oxidative balance score (OBS) is an indicator for assessing total oxidant status. This cross-sectional study aimed to investigate the relationships between OBS and factors associated with well-being among middle-aged and elderly women. Methods: Dietary habits, lifestyle factors, and factors associated with well-being, including physical, mental, and social health, were assessed based on the first-visit medical records in 385 Japanese women. The OBS was calculated using pro-oxidant factors (fat, saturated fatty acids, *n*-6 polyunsaturated fatty acids, iron, alcohol, smoking, body mass index, and waist circumference) and antioxidant factors (zinc, vitamins C, E, A, *n*-3 polyunsaturated fatty acids, genistein, and exercise). Results: After adjusting for age, menopausal status, and background factors, social engagement and weight-adjusted appendicular skeletal muscle mass (ASM/Wt) were found to be significantly associated with OBS (social involvement, odds ratio = 0.882, 95% confidence interval [CI] = 0.817–0.949, *p* = 0.001; ASM/Wt, odds ratio = 0.983, 95% CI = 0.974–0.990, *p* < 0.001). Conclusions: Higher oxidative stress was associated with a low level of social involvement and ASM/Wt. These findings indicate that oxidative balance is linked to social and physical aspects of well-being among middle-aged and older women. However, because of the cross-sectional design, causal relationships cannot be inferred, and the possibility of reverse causation should be considered.

## 1. Introduction

Declining estrogen levels are accompanied by increased oxidative stress in middle-aged and older women, particularly during the menopausal transition. Estrogen has potent antioxidant properties and plays a critical role in maintaining cardiovascular, neuropsychological, and musculoskeletal health. Consequently, estrogen deficiency has been associated with an increased risk of cardiovascular diseases (CVDs), metabolic syndrome, osteoporosis, sarcopenia, and neuropsychiatric disturbances such as depression and Alzheimer’s disease [1,2,3,4,5,6].

Chronic oxidative stress contributes to physical dysfunction and is associated with mental disorders including anxiety and depressive symptoms, fatigue, and reduced motivation, which may collectively impair social participation and overall well-being [7]. Physical function and physical activity are closely interrelated with social engagement; reductions in muscle mass and strength are associated with decreased mobility, fewer opportunities for social interaction, and lower quality of life in middle-aged and older women [8]. However, few studies have examined biological markers that may underlie both physical and psychosocial aspects of well-being in this population.

The oxidative balance score (OBS) is a composite index derived from dietary and lifestyle factors that reflect the balance between pro-oxidant and antioxidant exposures [9]. Higher OBS values indicate lower levels of oxidative stress. Previous studies have reported associations between OBS and several health outcomes, including cancer, metabolic syndrome, sarcopenia, and depression [9,10,11]. However, OBS calculation methods have not been standardized [12], and evidence regarding its association with skeletal muscle indices and social participation in middle-aged and older women remains scarce. Only a limited number of studies have explored relationships between OBS and conditions such as osteoporosis or urinary incontinence in women [13,14]. The present study aimed to comprehensively examine the associations between OBS and physical, psychological, and social well-being among middle-aged and older Japanese women using the OBS calculated based on previous research [9,12]. By elucidating these relationships, this study seeks to clarify the role of oxidative stress in linking biological vulnerability to functional and social outcomes in postmenopausal women.

## 2. Materials and Methods

### 2.1. Study Population

This cross-sectional analysis was conducted based on the first-visit medical records of 2065 Japanese women who enrolled in the Systematic Health and Nutrition Education Program (SHNEP) at the Menopause Clinic of the Institute of Science Tokyo from January 2008 to July 2023. All participants were referred to our clinic for the treatment of health problems. In the SHNEP, somatic and psychological health statuses are assessed using a variety of questionnaires and physical examinations to improve symptoms. In this study, the Menopausal Health Related Quality of Life Questionnaire (MHR-QOL) and the Hospital Anxiety and Depression scale (HADS) were used to evaluate the physical and mental symptoms. Dietary habits were assessed using the Brief-type Self-Administered Diet History Questionnaire (BDHQ), and participants’ physical fitness was assessed using a physical fitness analyzer. Study participants who had regular or no menstruation in the past 12 months were divided into pre- or natural menopause groups, respectively. Women who underwent bilateral oophorectomy before the onset of natural menopause were classified as the surgical menopause group. Women who experienced surgically induced menopause following hysterectomy and those who had missing age at menopause were excluded because of unknown menopause status. Participants who received systemic estrogen-containing menopausal hormone therapy were also excluded. The records of 385 study participants aged 40–69 years who completed questionnaires and underwent physical examinations were retrospectively analyzed. This analysis was exploratory in nature, and the analyses aimed to identify potential associations rather than to test predefined hypotheses.

The study was conducted in accordance with the Declaration of Helsinki, and the protocol was approved by the Institute of Science Tokyo Review Board (approval number, 774) on 23 March 2010. Informed consent for participation was obtained from all subjects involved in the study.

### 2.2. Questionnaire

History of noncommunicable diseases such as hypertension, dyslipidemia, and diabetes, and lifestyle factors, including regular exercise (yes/no) and frequency of smoking (none, <20, or ≥20 cigarettes per day), were assessed through medical interviews.

The BDHQ, a tool used to assess dietary habits, evaluates the dietary intake frequency of 61 foods, focusing on the typical Japanese diet during the previous month [15]. The daily intakes of 96 nutrient factors, after adjusting for total energy intake, were estimated using computer algorithms. The calculated daily consumption of foods and nutrients based on the BDHQ was previously verified after comparison with dietary records using a semi-weighed method [15,16]. Of the 96 nutrients, 43 with high validity were investigated in this study (Table S1). Nutrient intakes used for the OBS calculation were energy-adjusted.

The MHR-QOL, which was developed and validated in our clinic [17,18,19], is a modification of the Women’s Health Questionnaire and others [20,21,22]. It comprises four domains: physical health, mental health, life satisfaction, and social involvement. Each category has 9, 12, 5, and 12 items that are scored using a 4-point Likert scale or binary scale (Table S2). The sum of the scores for each item in the four categories was calculated. Furthermore, in the physical and mental health domains, eight symptoms (nausea, dizziness, numbness, muscle pains or joint pains, fatigue, headache, frequent urination, forgetfulness) were scored according to symptom frequency (none = 0 points, 1–2 times per week = 1 point, 3–4 times per week = 2 points, almost every day = 3 points). Vasomotor and insomnia symptoms scores were given as a combined score for the two items, with each assessment on a scale of 0–6 points. Somatic and neuropsychiatric symptoms were calculated by combining the scores related to the respective questionnaire items (somatic symptoms: 9 items; neuropsychiatric symptoms: 12 items).

The HADS, a reliable screening tool for anxiety and depression, was developed to evaluate the psychological status of patients with physical symptoms [23]. It consists of seven items each for anxiety and depression (Table S3). Participants respond to these items using a 4-point Likert scale. Those who received scores of 8–10 and 11–21 were considered likely or definitely experiencing anxiety or depression, respectively.

### 2.3. Physical Assessment

Height, weight, and waist circumference were measured, and body mass index (BMI) was calculated. Physical fitness, including body fat mass, lean body mass, muscle mass, and basal metabolism, was assessed using a bioimpedance analyzer (MC190-EM; Tanita, Tokyo, Japan). Furthermore, we calculated height-adjusted appendicular skeletal muscle mass (ASM/Ht^2^, kg/m^2^), weight-adjusted ASM (ASM/Wt, %), and fat mass-adjusted ASM (ASM/FM, %) to evaluate muscle mass.

Physical fitness tests were conducted to assess muscle strength, reaction, and flexibility. Handgrip strength was measured twice for each hand using a hand dynamometer (Yagami, Nagoya, Japan), and the average handgrip strength was calculated with the greater value of the two measurements. The reaction time in the ruler drop test was measured using a 60 cm wooden ruler weighing 110 g (Yagami). Seated participants, with their arms fixed on a desk and their fingers outstretched over the edge of the desk, attempted to catch a ruler suspended between their thumb and index fingers by its bottom end as quickly as possible when it was dropped. The scale on the ruler where the participants gripped was recorded. After the test was repeated seven times, the smallest and largest values were omitted, and the average reaction time (cm) was calculated using the remaining five values. The sit-and-reach test was used to assess flexibility, using a sit-and-reach box (Yagami).

### 2.4. Oxidative Balance Score

Pro-oxidant and antioxidant factors derived from dietary and lifestyle habits were identified based on descriptions from previous studies [9,12]. Iron, fat, saturated fatty acids, *n*-6 fatty acids, and alcohol were considered pro-oxidants, whereas zinc, *n*-3 fatty acids, vitamins C, E, and A, and genistein were considered antioxidants (Table S4). In addition, pro-oxidant factors included BMI, waist circumference, and smoking, whereas antioxidant factors included exercise habits. Based on the previous literature, each component of the OBS was weighted equally. Nutrient intake was divided into tertiles, and scores of 0–2 were assigned to the intake of pro-oxidants (low = 2; intermediate = 1, high = 0) or antioxidants (low = 0; intermediate = 1, high = 2). The scores for non- or mild drinkers (<10 g/day), moderate (10≤ and <20 g/day), and heavy drinkers (≥20 g/day) were rated 2, 1, and 0 points, respectively. BMI and smoking were assigned 0–2 points as follows: BMI—obese (≥30 kg/m^2^) = 0, overweight (25≤ and <30 kg/m^2^) = 1, non-obese (<25 kg/m^2^) = 2; smoking—heavy smokers (≥20 cigarettes per day) = 0, non-heavy smokers (0≤ and <20 cigarettes per day) = 1, non-smoker = 2. Study participants with a higher (≥90 cm) or lower (<90 cm) waist circumference received 0 or 1 point, respectively. Regular exercise habits were scored as 0 or 1 point for no or yes, respectively. OBS was calculated as the total number of points for each component, and a higher OBS indicated a lower oxidative stress status.

### 2.5. Statistical Analysis

Continuous variables are presented as median (interquartile range; IQR). Differences among the three groups were analyzed using the Kruskal–Wallis test or Fisher’s exact test. Multicollinearity among independent variables was formally assessed using variance inflation factors (VIF). A VIF value greater than 10 was considered indicative of problematic multicollinearity. The relationships between OBS and explanatory factors that differed significantly among the three groups were examined using multivariate logistic regression analysis after adjusting for background characteristics. Variables included in the regression models were selected based on (1) theoretical relevance from the prior literature, (2) significant differences in descriptive analyses, and (3) the absence of multicollinearity. Age, menopausal status, and noncommunicable diseases were selected as covariates in the adjusted model. Statistical analyses were performed using GraphPad Prism version 9 (GraphPad Software, San Diego, CA, USA) and JMP Pro 18 (SAS Institute Inc., Cary, NC, USA).

## 3. Results

The median age (IQR) of 385 participants was 51 (48–53) years. The background characteristics of the study participants based on OBS tertiles are shown in Table 1. Premenopausal and postmenopausal women accounted for 57.7% and 42.3% participants, respectively (natural 36.6%, surgical 5.7%). The group with high OBS (lower oxidative stress) had a higher proportion of postmenopausal women than the other groups (low/middle/high OBS: 38.2/39.4/51.4%).

Differences in physical and psychosocial health statuses and physical fitness among the OBS tertiles were assessed separately. Variables that are components of the OBS—such as smoking status, exercise, BMI (including weight), and waist circumference—are presented descriptively in Table 1, as differences in these variables across OBS tertiles are expected based on the construction of the index and therefore have no analytical implications. Analyses of variables that are not included in the OBS calculation showed significant differences among OBS tertiles in nausea and vasomotor symptom scores, life satisfaction, social involvement, body fat percentage, fat mass, appendicular and trunk muscle mass, basal metabolism, ASM/Ht^2^, ASM/Wt, ASM/FM, and sit-and-reach test performance (Table 1). Subsequently, OBS was divided into a low-OBS group (indicating high oxidative stress) and a high-OBS group (indicating low oxidative stress) based on the median value. This binary variable was used as the dependent variable in the logistic regression models (coded as 1 = low OBS and 0 = high OBS). Therefore, an odds ratio below 1 indicates that higher levels of the independent variable are associated with a lower likelihood of having low OBS, meaning a higher likelihood of having high OBS (reflecting lower oxidative stress). We investigated the associations between OBS and these selected factors using multivariate logistic regression analysis. Multicollinearity was addressed by excluding body fat percentage, fat mass, and basal metabolism, which showed high intercorrelations. For the remaining independent variables, variance inflation factors (VIF) ranged from 1.1 to 9.8, indicating no problematic multicollinearity. The associations with OBS were examined separately for somatic and psychosocial health statuses and physical fitness. OBS was significantly associated with social involvement (odds ratio [OR] 0.883; 95% confidence interval [CI], 0.811–0.959; *p* = 0.004) and ASM/Wt (OR 0.981; 95% CI 0.963–0.999; *p* = 0.033) (Table 2). After adjusting for age, menopausal status (Model 1), and noncommunicable diseases (hypertension, dyslipidemia, and diabetes mellitus) (Model 2), significant associations among OBS, social involvement (Model 2, OR 0.882; 95% CI 0.817–0.949; *p* = 0.001), and ASM/Wt (Model 2, OR 0.983, 95% CI 0.974–0.990, *p* < 0.001) remained (Table 3). Furthermore, the high-OBS group (low oxidative stress) had significantly higher levels of social engagement and ASM/Wt than the low-OBS group (Table 4).

## 4. Discussion

In this cross-sectional analysis, significant positive correlations were found among OBS, social involvement, and ASM/Wt, indicating that higher levels of oxidative stress were associated with lower levels of social engagement and ASM/Wt.

A large body of evidence supports the effect of social isolation on mortality. In a meta-analysis of 70 studies, both objective and subjective social isolation were associated with the risk of premature mortality. Furthermore, participants aged < 65 years had a higher risk of mortality than older participants [24]. A meta-analysis of 90 prospective cohorts by Wang et al. [25] demonstrated that social isolation increases the risk of all-cause, cancer, and CVD mortality. Loneliness has also been shown to affect mortality [24,25]. The American Heart Association provided a Scientific Statement in 2022 stating that social isolation and loneliness have adverse effects on the heart and brain health. The mechanisms underlying the increased risk of CVD involve multiple pathways, including dysregulation of the hypothalamic–pituitary–adrenal (HPA) axis, activation of the sympathetic nervous system (SNS), immune system alterations, oxidative stress, and behavioral and psychological changes [26,27]. Social isolation and loneliness lead to overactivation of the HPA axis and SNS. Glucocorticoid overproduction and resistance through HPA axis hyperactivity results in chronic inflammation and decreased endothelial nitric oxide production. Overactivation of the SNS and the resulting catecholamine release contribute to reduced cardiac output and increased total peripheral resistance. These complex, intertwined factors enhance inflammation and oxidative stress, leading to the development of atherosclerosis, hypertension, and CVDs. Oxidative stress may be essential for social isolation-induced HPA axis activation in the brain and may play a key role in the progression of CVDs [28]. Social isolation and loneliness are also related to changes in health behaviors, such as disrupted sleep, smoking, and physical inactivity. Middle-aged and older women may be particularly vulnerable to social isolation and loneliness due to age-related changes in health status and life circumstances, such as the transition from child-rearing to caregiving roles or the loss of close relationships. In this study, our findings indicate that lower levels of social involvement were associated with higher oxidative stress. However, because of the cross-sectional design, the direction of these associations cannot be determined. It is also possible that poorer health or lower muscle mass may reduce social participation and influence dietary quality. Longitudinal studies are needed to clarify these relationships.

ASM/Ht^2^ is generally used as a criterion for sarcopenia. Muscle mass is normally correlated with body size; thus, muscle mass is evaluated using body size-adjusted muscle masses, such as ASM/Ht^2^, ASM/Wt, and ASM/BMI. However, the differences among these indices remain controversial. A study investigating the differences between ASM/Ht^2^ and ASM/Wt in 1801 healthy Japanese individuals aged 18–90 years revealed that ASM/Ht^2^ was more strongly associated with osteoporosis risk factors (bone mineral density), while ASM/Wt was more strongly associated with CVD risk factors (triglycerides and high-density lipoprotein cholesterol) [29]. Additionally, in women, the ASM/Wt was inversely correlated with systolic and diastolic blood pressure. In the present study, we observed a positive relationship between ASM/Wt and OBS, which is known to be associated with CVDs risk. Several studies have shown an association between OBS and physical fitness. Xu W et al. detected that OBS was related to the prevalence of sarcopenia in 6677 US adults aged 20–59 years [11]. Moreover, in a national cohort study of the US population, OBS was positively correlated with ASM/BMI and handgrip strength [30]. There is an increased risk of sarcopenia, a progressive skeletal muscle disorder with accelerated loss of muscle mass, strength, and function, in postmenopausal women due to a lack of estrogen. Estrogen contributes to muscle health by promoting muscle regeneration through the activation and proliferation of muscle stem cells [31]. Furthermore, the anti-inflammatory and antioxidative effects of estrogen on skeletal muscles could prevent the progression of sarcopenia [31]. The results of this study suggest that higher oxidative stress is associated with lower muscle mass in middle-aged and older women. However, because this is a cross-sectional study, it is necessary to consider the possibility that reduced muscle mass and poor health status may influence oxidative balance.

The present study was limited by its small and narrowly defined population, making it difficult to generalize the findings. Participants were women attending a menopause clinic with pre-existing health problems, introducing a risk of selection bias. Causal relationships cannot be inferred due to the cross-sectional design, and the findings should be interpreted cautiously, particularly given the exploratory nature of the analyses and the absence of correction for multiple comparisons. Although the correlation between OBS and ASM/Wt was low (|R| < 0.3), this association warrants careful interpretation because OBS includes BMI and waist circumference, raising the possibility of mathematical coupling. To address this concern, we conducted a sensitivity analysis using a modified OBS that excluded BMI and waist circumference. The associations between OBS and both social involvement and ASM/Wt remained significant when using this alternative index, suggesting that the observed relationship is unlikely to be explained solely by body size-related components and may instead reflect an independent association.

Moreover, BDHQ may not include all sources of nutrients, and recall bias may exist. We could not assess all factors with pro- and antioxidant potential, such as the intake of selenium, aspirin, and non-steroidal anti-inflammatory drugs. Factors such as socioeconomic level and pre-existing diseases, including depression and inflammatory conditions, were also not assessed. These unmeasured variables may influence both oxidative stress and the associated lifestyle or physical characteristics, potentially leading to residual confounding. In addition, although the OBS includes an “exercise” component, this item does not reflect the full spectrum of physical activity (such as intensity, duration, or objectively measured activity) and therefore cannot replace comprehensive physical activity monitoring. Finally, the weight related to the effect of each component of the OBS on the oxidative status could not be considered, as no established method for calculating the OBS currently exists. It remains unclear whether the OBS calculation method used in this study is appropriate for middle-aged and older Japanese women. The number of components in the OBS varied among previous studies, ranging from 3 to 20 components [9]. In our study, genistein—a soy isoflavone with strong antioxidant properties—was included as one of the 13 OBS components to reflect Japanese dietary patterns, where soy intake is characteristically high. Although genistein has not been incorporated into previous OBS formulations, we considered its inclusion appropriate for this population. To evaluate whether this population-specific component influenced the results, we conducted a sensitivity analysis using a modified OBS that excluded genistein. The associations between OBS and the outcomes remained consistent, indicating that the findings were not dependent on the inclusion of genistein and supporting the robustness of the OBS construct across alternative index specifications. Despite these limitations, this study is the first to demonstrate a significant association among OBS, social engagement, and ASM/Wt in middle-aged and older women. We simultaneously identified factors associated with OBS, including physical and psychosocial health and physical fitness. In addition, dietary intake of pro-oxidant and antioxidant nutrients was assessed using the BDHQ, which reflects typical Japanese dietary habits.

## 5. Conclusions

The OBS was positively related to the levels of social engagement and ASM/Wt in this population, suggesting that the modification of dietary and lifestyle habits may promote both social and muscle health. These findings indicate that oxidative balance is linked to social and muscle health among middle-aged and older women. However, because of the cross-sectional design, causal relationships cannot be inferred, and the possibility of reverse causation should be considered. Dietary and lifestyle habits may also influence oxidative balance; however, longitudinal studies are needed to clarify these relationships.

## Figures and Tables

**Table 1:** Comparison of participant characteristics among OBS tertiles.

	OBS				*p*-Value
	Total (8–24)	Low (8–15)	Middle (16–18)	High (19–24)	
	*n* = 385	*n* = 136	*n* = 142	*n* = 107	
Age (years)	51 (48, 53)	51 (48, 53)	50 (48, 53)	51 (48, 54)	0.298 ^a^
Menopausal status Pre-/natural/surgical menopause (%)	57.7/36.6/5.7	61.8/31.6/6.6	60.6/35.9/3.5	47.7/43.9/7.5	<0.001 ^b^
Lifestyle factors					
Smoking (%) None/fewer than 20/20 or more cigarettes per day	89.6/6.8/3.6	78.7/1.8/9.6	92.3/7.0/0.7	100/0/0	<0.001 ^b^
Exercise (%) Yes/No	42.9/57.1	27.9/72.1	43.0/57.0	61.7/38.3	<0.001 ^b^
Noncommunicable diseases (%)					
Hypertension	10.1	9.6	9.9	11.2	0.936 ^b^
Dyslipidemia	7.5	8.8	5.6	8.4	0.555 ^b^
Diabetes mellitus	1.8	2.9	2.1	0	0.249 ^b^
Factors of well-being					
Body composition					
Height (cm)	157.6 (153.8, 161.2)	157.1 (153.7, 160.6)	157.7 (154.1, 161.8)	158.2 (153.7, 161.3)	0.751 ^a^
Weight (kg)	52.6 (47.4, 59.2)	55.7 (50.8, 63.2)	51.5 (46.5, 57.1)	49.8 (45.8, 56.4)	<0.001 ^a^
BMI (kg/m^2^)	21.1 (19.2, 23.9)	22.8 (20.4, 25.6)	20.9 (19.0, 23.5)	20.6 (18.7, 22.3)	<0.001 ^a^
Waist (cm)	79.0 (73.0, 87.0)	84.0 (76.0, 92.0)	78.3 (72.0, 84.0)	76.5 (71.0. 81.0)	<0.001 ^a^
Body fat percentage (%)	28.5 (22.9, 34.1)	32.7 (26.3, 37.7)	27.3 (22.0, 32.7)	26.4 (21.7, 31.3)	<0.001 ^a^
Fat mass (kg)	14.9 (10.8, 20.0)	18.8 (13.2, 23.5)	14.3 (10.3, 18.7)	13.1 (9.9, 16.9)	<0.001 ^a^
Lean mass (kg)	37.8 (35.6, 39.9)	38.3 (36.2, 40.0)	37.9 (35.4, 40.1)	37.6 (35.0, 39.6)	0.235 ^a^
Appendicular muscle mass (kg)	16.1 (15.0, 17.1)	16.4 (15.3, 17.5)	16.0 (15.0, 17.0)	15.8 (14.7, 16.8)	0.010 ^a^
Trunk muscle mass (kg)	19.0 (18.2, 19.8)	19.3 (18.6, 20.0)	18.9 (18.0, 19.8)	18.8 (17.8, 19.7)	0.024 ^a^
Basal metabolism (MJ/day)	1.10 (1.02, 1.17)	1.12 (1.06, 1.18)	1.09 (1.01, 1.16)	1.07(0.10)	0.003 ^a^
Height-adjusted appendicular muscle mass (kg/m^2^)	6.46 (6.12, 6.88)	6.58 (6.24, 6.99)	6.46 (6.11, 6.81)	6.35 (5.97, 6.64)	0.001 ^a^
Weight-adjusted appendicular muscle mass (%)	30.3 (28.1, 32.4)	28.9 (27.1, 30.1)	30.7 (28.9, 32.9)	30.9 (29.5, 32.9)	<0.001 ^a^
Fat mass-adjusted appendicular muscle mass (%)	106.7 (82.2, 141.3)	89.1 (71.4, 120.0)	113.2 (86.1, 1.48)	119.4 (93.8, 150.2)	<0.001 ^a^
Physical fitness test					
Handgrip strength (kg)	25.5 (22.3, 28.3)	25.5 (22.8, 28.1)	24.8 (22.0, 28.2)	26.3 (22.5, 28.9)	0.342 ^a^
Ruler drop test (cm)	22.8 (20, 25.6)	23.0 (21.0, 25.6)	21.9 (19.1, 25.0)	22.4 (20.1, 25.9)	0.115 ^a^
Sit-and-reach test (cm)	36.0 (30.0, 43.0)	35.0 (29.8, 41.6)	36.0 (29.0, 42.0)	39.0 (31.0, 45.0)	0.031 ^a^
Menopausal Health-Related Quality of Life Questionnaire					
Nausea (0–3 points)	0 (0, 1)	0 (0, 1)	0 (0, 0)	0 (0, 0)	0.021 ^a^
Dizziness (0–3 points)	0 (0, 1)	0 (0, 1)	0 (0, 1)	0 (0, 1)	0.273 ^a^
Numbness (0–3 points)	0 (0, 1)	0 (0, 1)	0 (0, 1)	0 (0, 1)	0.896 ^a^
Muscle pains or joint pains (0–3 points)	3 (1, 3)	3 (1, 3)	3 (1, 3)	3 (1, 3)	0.781 ^a^
Fatigue (0–3 points)	3 (1, 3)	3 (1, 3)	3 (1, 3)	2 (1, 3)	0.302 ^a^
Headache (0–3 points)	1 (0, 1)	1 (0, 1)	1 (0, 1)	0 (0, 1)	0.192 ^a^
Frequent urination (0–3 points)	0 (0, 2)	0 (0, 2)	0.5 (0, 2)	0 (0, 2)	0.581 ^a^
Forgetfulness (0–3 points)	1 (0, 2)	1 (0, 2)	1 (0, 2)	1 (0, 2)	0.592 ^a^
Vasomotor symptoms score (0–6 points)	2 (0, 4)	2 (0.8, 4)	2 (0, 4.8)	1 (0, 3)	0.034 ^a^
Insomnia symptoms score (0–6 points)	2 (0, 4)	2 (0, 4)	2 (0, 4)	2 (0, 4)	0.888 ^a^
Somatic symptom score (0–27 points)	10 (6, 14)	10 (7,14)	10 (7,14)	9 (5.5, 13)	0.084 ^a^
Neuropsychiatric symptoms score (0–36 points)	11 (5, 20)	11 (6, 20)	12.5 (6, 20)	9 (4, 19)	0.312 ^a^
Life satisfaction (0–15 points)	7 (5, 9)	6 (5, 9)	7 (5, 9)	8 (5.5, 10)	0.034 ^a^
Social involvement (0–16 points)	9 (7, 11)	9 (7, 10)	9 (7, 11)	9 (8, 11)	0.005 ^a^
Hospital Anxiety and Depression Scale	15 (10, 20)	14.5 (10, 20)	16 (11, 20)	13 (8, 19.5)	0.204 ^a^
Anxiety subscale score	8 (5, 10)	8 (5, 10)	8 (5, 11)	7 (5, 10.5)	0.228 ^a^
Depression subscale score	7 (4, 10)	7 (4, 10)	8 (5, 10)	7 (3, 9)	0.209 ^a^

OBS, oxidative balance score; BMI, body mass index. Values are expressed as median (interquartile range) or as percentage. ^a^ Kruskal–Wallis test, ^b^ Fisher’s exact test. Note: Variables that are components of the OBS are presented for descriptive purposes only and are not interpreted analytically.

**Table 2:** Menopausal symptoms and physical fitness associated with OBS.

**Factors Of Well-Being**	**Odds Ratio**	**95% Confidence Interval**	***p*** **-Value**
Somatic health			
Nausea	0.912	0.707, 1.078	0.474
Vasomotor symptoms	0.943	0.856, 1.039	0.235
Life satisfaction	1.001	0.932, 1.078	0.959
Social involvement	0.883	0.811, 0.959	0.004
Physical fitness			
Appendicular muscle mass	1.050	0.700, 1.616	0.817
Trunk muscle mass	0.892	0.523, 1.479	0.665
Height-adjusted appendicular muscle mass	1.489	0.813, 2.751	0.200
Weight-adjusted appendicular muscle mass	0.981	0.963, 0.999	0.033
Fat mass-adjusted appendicular muscle mass	1.242	0.462, 2.954	0.634
Sit-and-reach test	0.989	0.968, 1.012	0.349

OBS, oxidative balance score.

**Table 3:** Relationships between OBS and social involvement and weight-adjusted appendicular muscle mass.

	Odds Ratio	95% Confidence Interval	*p*-Value
Social involvement			
Model 1	0.878	0.814, 0.945	0.001
Model 2	0.882	0.817, 0.949	0.001
Weight-adjusted appendicular muscle mass			
Model 1	0.982	0.975, 0.989	<0.001
Model 2	0.983	0.974, 0.990	<0.001

OBS, oxidative balance score. Model 1: adjusted for age and menopausal status; Model 2: adjusted for age, menopausal status, and noncommunicable diseases (hypertension, dyslipidemia, and diabetes mellitus).

**Table 4:** Relationships between OBS tertiles and social involvement and weight-adjusted appendicular muscle mass.

OBS Tertile	Odds Ratio	95% Confidence Interval	*p*-Value
Social involvement			
Model 1			
Low	Reference		
Middle	0.894	0.811, 0.983	0.022
High	0.863	0.782, 0.948	0.003
Model 2			
Low	Reference		
Middle	0.932	0.852, 1.017	0.117
High	0.869	0.786, 0.957	0.005
Weight-adjusted appendicular muscle mass			
Model 1			
Low	Reference		
Middle	0.982	0.973, 0.990	<0.001
High	0.975	0.964, 0.984	<0.001
Model 2			
Low	Reference		
Middle	0.982	0.974, 0.991	<0.001
High	0.975	0.964, 0.985	<0.001

OBS, oxidative balance score. Model 1: adjusted for age and menopausal status; Model 2: adjusted for age, menopausal status, and noncommunicable diseases (hypertension, dyslipidemia, and diabetes mellitus).

## Data Availability

Raw data were generated at the Institute of Science of Tokyo. Derived data supporting the findings of this study are available from the corresponding author MT on request.

## Supplementary Materials

The following supporting information can be downloaded at: https://www.mdpi.com/article/10.3390/nu18040688/s1, Table S1: Forty-three nutrients with high validity in the brief-type self-administered diet history questionnaire, Table S2: The Menopausal Health Related Quality of Life Questionnaire, Table S3: The Hospital Anxiety and Depression Scale, Table S4: Pro-oxidant and antioxidant factors.
