# Supplementary material for: Oxidative Balance Score Is Associated with Social Involvement and Weight-Adjusted Appendicular Skeletal Muscle Mass in Middle-Aged and Older Japanese Women
Source: Nutrients. 2026 Feb 20;18(4):688. doi: 10.3390/nu18040688 (PMC12943278; doi:10.3390/nu18040688)
Supplement: Supplementary file 1 [file nutrients-18-00688-s001.zip › nutrients-4120701-supplementary.pdf]

Table S1. Forty-three nutrients with high validity in the brief-type self-administered diet history questionnaire

| | Physical and psychological symptoms | Score | | | |
|---|---|---|---|---|---|
| | | None | Mild | Moderate | Severe |
| 1 | I feel nauseous. | 3 | 2 | 1 | 0 |
| 2 | I feel dizzy. | 3 | 2 | 1 | 0 |
| 3 | My limbs are numb. | 3 | 2 | 1 | 0 |
| 4 | I have backache, stiff neck, and joint pain. | 3 | 2 | 1 | 0 |
| 5 | I get tired easily. | 3 | 2 | 1 | 0 |
| 6 | I have headache. | 3 | 2 | 1 | 0 |
| 7 | I go to the restroom a lot. | 3 | 2 | 1 | 0 |
| 8 | I find hot flushes embarrassing. | 3 | 2 | 1 | 0 |
| 9 | I often sweat in my sleep. | 3 | 2 | 1 | 0 |
| 10 | I feel less enthusiastic about things than before. | 3 | 2 | 1 | 0 |
| 11 | Things I used to enjoy have become a bit of a chore. | 3 | 2 | 1 | 0 |
| 12 | I do less than I would like. | 3 | 2 | 1 | 0 |
| 13 | My mood is generally depressed. | 3 | 2 | 1 | 0 |
| 14 | I forget things easily. | 3 | 2 | 1 | 0 |
| 15 | I cannot concentrate easily. | 3 | 2 | 1 | 0 |
| 16 | I upset about small things. | 3 | 2 | 1 | 0 |
| 17 | I feel nervous about small things. | 3 | 2 | 1 | 0 |
| 18 | I am not content with my sexual life. | 3 | 2 | 1 | 0 |
| 19 | I have trouble falling asleep. | 3 | 2 | 1 | 0 |
| 20 | The quality of my sleep is low. | 3 | 2 | 1 | 0 |
| 21 | I am not content with my appearance. | 3 | 2 | 1 | 0 |
| | **Life satisfaction** | definitely false | probably false | probably true | definitely true |
| 1 | I am completely satisfied with my day-to-day work. | 0 | 1 | 2 | 3 |
| 2 | I feel full of energy. | 0 | 1 | 2 | 3 |
| 3 | I look forward with pleasure to the future. | 0 | 1 | 2 | 3 |
| 4 | Life is interesting and every day brings something new. | 0 | 1 | 2 | 3 |
| 5 | Life is a real pleasure. | 0 | 1 | 2 | 3 |

| | Social involvement | difinitely false | probably false | probably true | definitely true |
|---|---|---|---|---|---|
| 1 | Do you believe that it is important to get a lot of recognition in any organizations or clubs, such as parent-teacher organization, reunion, culture center, and women's committee? | 0 | 1 | 2 | 3 |
| 2 | Do you believe that it is important to socialize with the people living in the neighborhood community? | 0 | 1 | 2 | 3 |
| | | true | | false | |
| 3 | In your daily life, do you have use for the knowledge and skills you have acquired during your life? | 1 | | 0 | |
| 4 | Do you feel that you are of great importance to other people and that you have a useful function in society? | 1 | | 0 | |
| 5 | Are you a member of any organization or clubs, such as parent-teacher organization, reunion, and culture center? | 1 | | 0 | |
| 6 | Are you in any position of trust in any organizations or clubs? | 1 | | 0 | |
| 7 | Have you taken part in meetings of different organizations, clubs, etc., in the last year? | 1 | | 0 | |
| 8 | Have you taken an active part in the discussion at a meeting, taken part in a political meeting, and contacted a consumers' association in the last year? | 1 | | 0 | |
| 9 | Have you been to parties at your friends', relatives', and neighbors' home in the last year? | 1 | | 0 | |
| 10 | Have you actively addressed environmental conservation in the last year? | 1 | | 0 | |
| 11 | Is there anyone in your neighborhood from whom you can borrow things or with whom you can exchange services? | 1 | | 0 | |
| 12 | Is there anyone in your neighborhood from whom you can get help if you fall ill? | 1 | | 0 | |

Table S2. The Menopausal Health Related Quality of Life Questionnaire

| Nutrients | | | | | |
|---|---|---|---|---|---|
| Protein | Protein | Vitamin | Retinol | Ash | Ash |
| | Animal protein | | β-Carotene | Mineral | Sodium |
| | Vegetable protein | | Retinol equivalent | | Potassium |
| Carbohydrate | Carbohydrate | | Vitamin D | | Calcium |
| | Soluble dietary fiber | | α-Tocopherol | | Magnesium |
| | Insoluble dietary fiber | | Vitamin K | | Phosphorus |
| | Dietary fiber | | Vitamin $B_1$ | | Iron |
| Fat | Fat | | Vitamin $B_2$ | | Zinc |
| | Animal fat | | Niacin | | Copper |
| | Vegetable fat | | Vitamin $B_6$ | | Manganese |
| | Saturated fatty acid | | Vitamin $B_{12}$ | Others | Daidzein |
| | Monounsaturated fatty acid | | Folic acid | | Genistein |
| | Polyunsaturated fatty acid | | Pantothenic acid | | Alcohol |
| | Cholesterol | | Vitamin C | | |
| | n-3 fatty acid | | | | |
| | n-6 fatty acid | | | | |

Table S3. The Hospital Anxiety and Depression Scale

| Scale/item | Item text |
|---|---|
| A/1 | I feel tense or wound up |
| A/3 | I get a sort of frightened feeling as if something awful is about to happen |
| A/5 | Worrying thoughts go through my mind |
| A/7 | I can sit at ease and feel relaxed |
| A/9 | I get a sort of frightened feeling like 'butterflies' in the stomach |
| A/11 | I feel restless as if I have to be on the move |
| A/13 | I get sudden feeling of panic |
| D/2 | I still enjoy the things I used to enjoy |
| D/4 | I can laugh and see the funny side of tings |
| D/6 | I feel cheerful |
| D/8 | I feel as if I am slowed down |
| D/10 | I have lost interest in my appearance |
| D/12 | I look forward with enjoyment to things |
| D/14 | I can enjoy a good book or TV program |

Table S4. Pro-oxidant and antioxidant factors

| Pro-oxidant | Antioxidant |
|---|---|
| Iron | Zinc |
| Fat | n-3 fatty acid |
| Saturated fatty acid | Vitamin C |
| n-6 fatty acid | Vitamin E |
| Alcohol | Vitamin A |
| Smoking | Genistein |
| Body mass index | Exercise |
| Waist circumference | |
